# The effect on the extracellular matrix of the deep fascia in response to leg lengthening

**DOI:** 10.1186/1471-2474-9-101

**Published:** 2008-07-09

**Authors:** Hai-Qiang Wang, Xin-Kui Li, Zi-Xiang Wu, Yi-Yong Wei, Zhuo-Jing Luo

**Affiliations:** 1 Institute of Orthopaedics, Xijing Hospital, Fourth Military Medical University, Xi'an, People's Republic of China, 710032

## Abstract

**Background:**

Whereas the alterations of diverse tissues in cellular and molecular levels have been investigated during leg lengthening via microscopy and biochemical studies, little is known about the response of deep fascia. This study aims to investigate the changes of the extracellular matrix in deep fascia in response to leg lengthening.

**Methods:**

Animal model of leg lengthening was established in New Zealand white rabbits. Distraction was initiated at a rate of 1 mm/day and 2 mm/day in two steps, and preceded until increases of 10% and 20% in the initial length of tibia had been achieved. Alcian blue stain and picrosirius-polarization method were used for the study of the extracellular matrix of deep fascia samples. Leica DM LA image analysis system was used to investigate the quantitative changes of collagen type I and III.

**Results:**

Alcian blue stain showed that glycosaminoglycans of fascia of each group were composed of chondroitin sulphate and heparin sulphate, but not of keratan sulphate. Under the polarization microscopy, the fascia consisted mainly of collagen type I. After leg lengthening, the percentage of collagen type III increased. The most similar collagen composition of the fascia to that of the normal fascia was detected at a 20% increase in tibia length achieved via a distraction rate of 1 mm/d.

**Conclusion:**

The changes in collagen distribution and composition occur in deep fascia during leg lengthening. Although different lengthening schemes resulted in varied matrix changes, the most comparable collagen composition to be demonstrated under the scheme of a distraction rate of 1 mm/day and 20% increase in tibia length. Efficient fascia regeneration is initiated only in certain combinations of the leg load parameters including appropriate intensity and duration time, e.g., either low density distraction that persist a relatively short time or high distraction rates.

## Background

The concept of distraction histogenesis was introduced by G.A.Ilizarov and classic papers were published in the English literature in 1989[1,2]. Gradual traction on living tissues creates stresses that can stimulate regeneration and maintain active growth of certain tissue structures. Ilizarov designated this principle the Law of Tension-Stress [1]. The clinical applications of this principle in orthopaedics include limb lengthens discrepancy or short stature [3,4], delayed unions and nonunions of fractures [5], limb deformities correction [6], congenital pseudoarthrosis [7], and the treatment of bone defect [8]. The basic research of limb lengthening falls into two aspects. One is the responses of various tissues under tension stress during limb lengthening, including new bone formatted in the distraction gap, muscles, tendons, vessels and nerves [9-11]. The other concerns the underlying molecular mechanism of this principle [12,13]. However, among all the previous investigations, reports related to the deep fascia remain limited.

Congenital and developmental deformities are intriguing and difficult to overcome for orthopedic surgeons. These deformities are closely related to the lesions of soft tissues, mainly dense connective tissue. The deep fascia, belonging to connective tissue in histology, is just one of the most directly related tissues. In the previous study, histological and ultrastructural alterations of deep fascia in response to leg lengthening have been reported [14]. However, the response of extracellular matrix of fascia remains unknown. The aim of this study is to investigate the morphological characteristics of extracellular matrix, including glycosaminoglycans (GAGs) and extracellular matrix proteins, of deep fascia during leg lengthening and to evaluate candidate strategies to improve regeneration of fascia tissues, i.e., distraction fasciogenesis.

## Methods

### Establishment of leg lengthening animal model

In 24 adult New Zealand white rabbits (License number SCXK 2002–005, lab animal center of the Fourth Military Medical University), the fascia of the leg was distracted by a unilateral external fixator applied with four pins to the medial surface of the tibia. Adult rather than immature rabbits were used to eliminate the factors of growth and development which may affect the accuracy of the study. The committee on animal experimentation of Fourth Military Medical University approved all experiments, which met the NIH guidelines for the care and use of laboratory animals. A monofocal proximal diaphysis osteotomy between the second and the third pins was performed with just little incisions. Then the periosteum and the skin were closed [15,16].

### Leg lengthening

Seven days after operation [17,18], axial distraction was conducted at 2 different rates, 1 and 2 mm per day, respectively. Lengthening was performed twice daily until 10% and 20% increases in the initial length of the tibia had been achieved. The initial length of the tibia varied between each rabbit with an average length of 9 centimeters. Thus the lengthening values were correspondingly 0.9 and 1.8 centimeters. 24 adult New Zealand white rabbits were randomly divided into 4 groups. Each group included 6 animals. The animals were grouped as indicated in Table 1. In a sham group of 2 animals, the external fixator system was applied and osteotomy was made, but no lengthening was performed.

**Table 1:** Classification of animals

**Increases in length of tibia**		**Lengthening**	**rate**
	
		1 mm/day	2 mm/day
10%		Group A	Group C
20%		Group B	Group D

### Histochemistry

The fascia with attached muscles of different groups was fixed in 10% neutral buffered formalin and embedded in paraffin. The approximate size of the biopsies was 1 × 0.5 centimeters. Longitudinal and cross sections were randomly selected from the fascia systematically. Sections were stained with Alcian blue 8GX(Sigma Chemical Company). To distinguish between sulfated and nonsulfated GAGs, sections were stained to equilibrim in ascending molarities of magnesium chloride(MgCl_2_) in a buffed Alcian blue solution, pH 5.8, according to the critical electrolyte concentration method of Scott and Dorling [19]. At low molarities of magnesium chloride (MgCl_2_), *e.g*., 0.06 M, both carboxylated and sulfated polyanionic groups of proteoglycans were stained, whereas at higher molarities (0.3, 0.5, 0.7, 0.9 M) only sulfated groups were stained. Picrosinus-polarization method was used to distinguish collagen type I from type III. Leica DM LA automatic microsystem and Leica Qwin V3.2 software (Leica Microsystem Ltd., Germany) were used to study the percentage of collagen type I and type III in the matrix. In the fascia sections, pixels corresponding to different colors were evaluated by the software and the color information was transformed to quantitative values and comparative percentages.

### Statistic analysis

Statistical comparisons were carried out using SPSS (SPSS, Chicago). First, the data were screened to detect outliers. Using origin 5.0 software, the data were evaluated by analysis of variance and followed by Student t test. A *P *value of less than 0.05 was considered significant.

## Results

Alcian blue stain showed that GAGs of fascia of each group were composed of chondroitin sulphate and heparin sulphate, but not of keratan sulphate.

### The morphology of normal deep fascia

#### Microscopy

Under the polirization microscopy, the picrosinus staining revealed that the fascia consisted mainly of collagen type I (red staining) as collagen type III (yellow) was rarely detected.

### The morphology of deep fascia after leg lengthening

#### Microscopy

**Figure 1:**
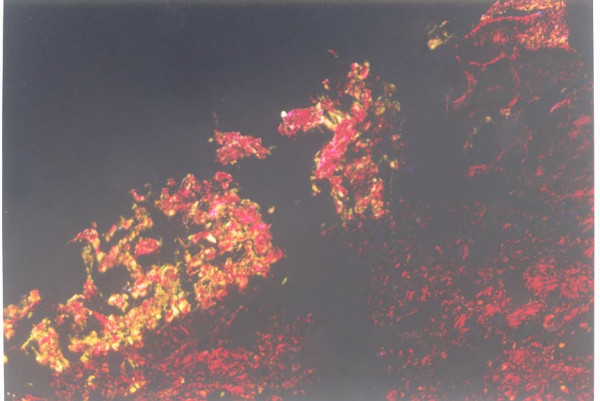
Fascia distracted at 2 mm/d with 10% increase in tibia length under the polarization microscopy (Original magnification 10 × 10). A high abundance and a wide distribution of collagen type II (yellow) were detected.

**Figure 2:**
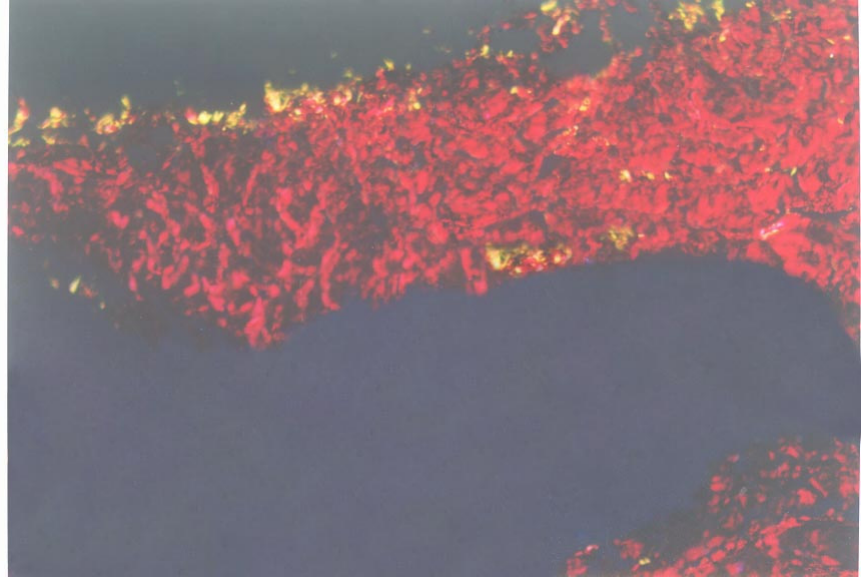
Fascia distracted at 1 mm/d with 10% increase in tibia length under the polarization microscopy (Original magnification 10 × 10). The deep fascia mainly consisted of collagen type I (red), and a basal collagen type II (yellow) distribution was detected in layers D1 and D2.

In contrast to comparable percentages of collagen type I in the matrixes of control and distraction group, the picrosinus staining showed that the relative abundance of collagen type III increased dramatically in the deep fascia matrix of rabbits subjected to 2 mm/day distraction (group C and D, Fig 1). A much lower but significant increase in collagen type III level was also observed in the fascia following continuous 1 mm/day distraction until 10% leg lengthening (group A, Fig 2). The matrix composition assay revealed slightly decreased percentages of collagen type I in groups C and D, which might result from relatively elevated collagen type III levels. Collagen type III was mainly distributed in layers D1 and D2. Given that 3 layers, including 2 dense layers and 1 loose layer, were defined in deep fascia microscopy, layers D1 and D2 refers to the microscopic dense layer of deep fascia, as demonstrated in a previous study [14]. The relative abundances of collagen type I and III of fascia of each group were shown in table 2.

**Table 2:** Relative abundances of collagens in fascia matrix ($\bar{\text{x}}$ ± s)

Group	Composition percentage	
	
	Type I	Type III
Normal	97.71 ± 0.69	2.28 ± 0.69
A	97.02 ± 0.40*	3.15 ± 0.14**
B	97.62 ± 0.61*	2.41 ± 0.63*
C	86.88 ± 2.41**	13.14 ± 2.13**
D	93.54 ± 0.60**	6.57 ± 0.75**

*P > 0.05, **P < 0.05.

## Discussion

The distribution and composition of extracellular matrix in tissues play important roles in the etiology, pathology and mechanism of diseases. In particular, the abundance alterations of collagens are closely related to the injury and repairing of tissues, fibrosis pathology and the physiologic process of tissue regeneration. The total amount of collagen type I is approximately equal to that of collagen type III under normal conditions in most organs and tissues. Total collagens increases from 4% in normal liver to 10% in cirrhosis, and the levels of collagen type I reaches 4 times collagen type III as cirrhosis occurs [20]. Similarly, collagen type I increases dramatically while collagen type III decreases in the fibrosis of lung [21]. In contrast, collagen type III but not collagen type I level increases during tissue repair, suggesting a prior role of collagen type III in the regeneration of related tissues.

In this study, a distraction rate of 2 mm/d led to injuries of fascia and increasing amount of collagen type III in the matrix, which represents a repairing process of related tissues. The closest abundance of collagen to that of normal fascia was detected in the matrix of fascia that had been distracted at a rate of 1 mm/d until 20% increase in tibia length was achieved. Combined with our previous findings of increased amount of reticular fibers and ribosome, a nuclear split, the activation of endotheliocyte and newly formed young collagenous fibrils in the same scheme [14], these data indicate that the regeneration of multi-tissues involving deep fascia occurs in animals subjected to distraction-forced leg lengthening. While the distraction rates represent a certain load exerted on legs, the increments in tibia length (10% and 20%) may reflect the duration time of the load. As a result, efficient fascia regeneration is initiated only in certain combinations of the leg load parameters including appropriate intensity and duration time, e.g., either low density distraction that persist a relatively short time or high distraction rates. This may explain why 20% lengthening at a rate of 1 mm per day causes less collagen damage than 10%. Whereas GAGs are essential components of the extracellular matrix in normal, embryonic, or tumor tissues, in our study, a strong staining with Alcian blue in both control and distracted fascias indicated the presence of chondroitin sulphate and heparin sulphate, but not of keratan sulphate. However, the accurate detection of GAGs in biological samples has been precluded by the lack of sensitive methods [22].

Histochemical assays, including Alcian blue stain and picrosinus-polarizition method, are commonly used in the studies of the extracellular matrix, and computer-assisted imagine analysis system is usually imperative for a quantitative analysis of the imaging data. In this study to dissect the matrix alterations following leg lengthening, sections of different fascia regions were prepared, and pixels corresponding to different colors are analyzed to quantify the relative abundances of the major matrix components, collagen types I and III. Available techniques to detect GAGs usually require dissociative extraction of tissues [23]. For histochemistry, Alcian blue staining is generally used, in combination with critical electrolyte conditions at a definite pH [19]. The presence of sulfated GAGs can be demonstrated in the deep fascia using Alcian blue added MgCl_2_. Due to currently unavailable quantification methods, the measurement of structural constituents on sections is usually difficult and the results are often doubtful [24]. Further study focusing on more sensitive histochemical methods to quantify GAGs may facilitate the quantitative comparison of extracelular matrix during leg lengthening.

Together, this study investigated the extracellular matrix changes including GAGs and the distribution and abundance of collagens in deep fascia in the context of leg lengthening for the first time according to our knowledge. Although further studies are definitely needed to dissect the synergized cellular and extracellular matrix signaling network responsible for regeneration and repair of the deep fascia, our study provides evidence for a potential clinical application of the Tension-Stress principle to deformity correction and limb lengthening.

## Competing interests

The authors declare that they have no competing interests.

## Authors' contributions

ZJL and HQW conceived of the study, participated in the design of the study and performed the statistical analyses. All authors carried out the experiments. HQW drafted the manuscript with the help of ZXW and YYW. All authors have read and approved the final manuscript.

## Pre-publication history

The pre-publication history for this paper can be accessed here:



## References

[B1] Ilizarov GA (1989). The tension-stress effect on the genesis and growth of tissues. Part I. The influence of stability of fixation and soft-tissue preservation. Clin Orthop.

[B2] Ilizarov GA (1989). The tension-stress effect on the genesis and growth of tissues. Part II. The influence of the rate and frequency of distraction. Clin Orthop.

[B3] Aldegheri R, Dall'Oca C (2001). Limb lengthening in short stature patients. J Pediatr Orthop B.

[B4] Aldegheri R (1999). Distraction osteogenesis for lengthening of the tibia in patients who have limb-length discrepancy or short stature. J Bone Joint Surg Am.

[B5] Pullen C, Manzotti A, Catagni MA, Guerreschi F (2003). Treatment of post-traumatic humeral diaphyseal nonunion with bone loss. J Shoulder Elbow Surg.

[B6] Wallander H, Hansson G, Tjernstrom B (1996). Correction of persistent clubfoot deformities with the Ilizarov external fixator. Experience in 10 previously operated feet followed for 2–5 years. Acta Orthop Scand.

[B7] Paley D, Catagni M, Argnani F, Prevot J, Bell D, Armstrong P (1992). Treatment of congenital pseudoarthrosis of the tibia using the Ilizarov technique. Clin Orthop.

[B8] Robert RS, Weitzman AM, Tracey WJ, Freudigman P, Katz HV, Ilizarov S (2006). Simultaneous treatment of tibial bone and soft-tissue defects with the Ilizarov method. J Orthop Trauma.

[B9] Aldegheri R, Renzi-Brivio L, Agostini S (1989). The callotasis method of limb lengthening. Clin Orthop.

[B10] Polo A, Aldegheri R, Zambito A, Trivella G, Manganotti P, De Grandis D, Rizzuto N (1997). Lower-limb lengthening in short stature. An electrophysiological and clinical assessment of peripheral nerve function. J Bone Joint Surg Br.

[B11] Polo A, Zambito A, Aldegheri R, Tinazzi M, Rizzuto N (1999). Nerve conduction changes during lower limb lengthening. Somatosensory evoked potentials (SEPs) and F-wave results. Electromyogr Clin Neurophysiol.

[B12] Rauch F, Lauzier D, Croteau S, Travers R, Glorieux FH, Hamdy R (2000). Temporal and spatial expression of bone morphogenetic protein-2, -4, and -7 during distraction osteogenesis in rabbits. Bone.

[B13] Rhee ST, Buchman SR (2005). Colocalization of c-Src (pp60src) and bone morphogenetic protein 2/4 expression during mandibular distraction osteogenesis: in vivo evidence of their role within an integrin-mediated mechanotransduction pathway. Ann Plast Surg.

[B14] Wang HQ, Li MQ, Wu ZX, Zhao L (2007). The deep fascia in response to leg lengthening with particular reference to the Tension-Stress principle. J Pediatr Orthop.

[B15] Frierson M, Ibrahim K, Boles M, Bote H, Ganey T (1991). Distraction osteogenesis. A comparison of corticotomy techniques. Orthop Clin North Am.

[B16] Yasui N, Kojimoto H, Shimizu H, Shimomura Y (1991). The effect of distraction upon bone, muscle, and periosteum. Orthop Clin North America.

[B17] White SH, Kenwright J (1990). The timing of distraction of an osteotomy. J Bone Joint Surg Br.

[B18] Yasui N, Kojimoto H, Sasaki K, Kitada A, Shimizu H, Shimomura Y (1993). Factors affecting callus distraction in limb lengthening. Clin Orthop.

[B19] Scott JE, Dorling J (1965). Differential staining of acid glycosaminoglycans (mucopolysaccharides) by alcian blue in salt solutions. Histochemie.

[B20] Diegelmann RF, Guzelian PS, Gay R, Gay S (1983). Collagen formation by the hepato cyte in primary monolayer culture and *in vivo*. Sci.

[B21] Madri JA, Furchmayr H (1980). Collage polymorphism in the lung. An immunochemical study of pulmonary fibrosis. Human Pathology.

[B22] Yoon JH, Brooks R, Halper J (2002). Immunoblotting assays for keratan sulfate. Anal Biochem.

[B23] Gigard N, Delpech A, Delpech B (1986). Characterization of hyaluronic acid on tissue sections with hyaluronectin. J Histochem Cytochem.

[B24] Weibei ER, Kistler GS, Scherle WF (1966). Practical stereological methods for morphometric cytology. J Cell Biology.

